# Greater accuracy of radiomics compared to deep learning to discriminate normal subjects from patients with dementia: a whole brain 18FDG PET analysis

**DOI:** 10.1097/MNM.0000000000001810

**Published:** 2024-02-19

**Authors:** Alberto Bestetti, Luigi Calabrese, Vincenzo Parini, Carla Fornara

**Affiliations:** a Department of Clinical and Community Sciences, State University of Milan, Sesto San Giovanni,; b Nuclear Medicine Department, MultiMedica Hospital,; c Radiation Oncology Department, MultiMedica Hospital and; d Division of Neurology, MultiMedica Hospital, Sesto San Giovanni, Italy

**Keywords:** deep learning, dementia, FDG PET, radiomics

## Abstract

Objective FDG PET imaging plays a crucial role in the evaluation of demented patients by assessing regional cerebral glucose metabolism. In recent years, both radiomics and deep learning techniques have emerged as powerful tools for extracting valuable information from medical images. This article aims to provide a comparative analysis of Radiomics and 3D-deep learning (CNN) approaches in the evaluation of 18F-FDG PET whole-brain images in patients with dementia and normal controls.

**Methods:**

18F-FDG brain PET and clinical score were collected in 85 patients with dementia and 125 healthy controls (HC). Patients were assigned to various form of dementia on the basis of clinical evaluation, follow-up and voxels comparison with HC using a two-sample Student’s *t*-test, to determine the regions of brain involved. Radiomic analysis was performed on the whole brain after normalization to an optimized template. After feature selection using the minimum redundancy maximum relevance method and Pearson’s correlation coefficients, a Neural Network model was tested to find the accuracy to classify HC and demented patients. Twenty subjects not included in the training were used to test the models. The results were compared with those obtained by conventional CNN model.

**Results:**

Four radiomic features were selected. The validation and test accuracies were 100% for both models, but the probability scores were higher with radiomics, in particular for HC group (*P* = 0.0004).

**Conclusion:**

Radiomic features extracted from standardized PET whole brain images seem to be more accurate than CNN to distinguish patients with and without dementia.

## Introduction

Dementia, including Alzheimer’s disease (AD) and other neurodegenerative disorders, poses significant diagnostic challenges due to its complex and heterogeneous nature [1]. FDG PET imaging offers valuable insights into the functional alterations of the brain by measuring regional cerebral glucose metabolism. Radiomics and deep learning techniques provide complementary approaches for analyzing FDG PET images, enabling quantitative and automated evaluation [2]. Deep learning techniques, particularly convolutional neural networks (CNNs), have shown remarkable performance in image analysis tasks. When applied to FDG PET images, deep learning models can learn complex patterns and relationships directly from the data without relying on predefined features. CNNs can be trained to classify dementias, identify biomarkers, and detect subtle changes in FDG uptake patterns, offering high accuracy and efficiency in the evaluation process [3–6]. The majority of these studies for AD diagnosis were conducted on MRI scans. Liu *et al*. [3] used multiple deep 3D-CNN on different local image patches to learn the discriminative features of MRI and FDG PET images, obtained from Alzheimer’s Disease Neuroimaging Initiative (ADNI) dataset. The classification performance for AD/HC diagnosis was 93.26%. Radiomics involves the extraction of a vast array of quantitative features from medical images, encompassing various aspects such as intensity, shape, texture, and spatial relationships. In the context of FDG PET, radiomic analysis can provide valuable information about metabolic patterns, heterogeneity, and regional changes in glucose metabolism. These quantitative features have the potential to aid in differentiating dementias, predicting disease progression, and evaluating treatment response [7–9]. Yupeng Li *et al*. [9] assessed FDG PET and clinical cognitive scales in AD, MCI (Mild Cognitive Impairment) and HC subjects, from ADNI cohorts. They determined the brain region of interest involved (ROIs), comparing, by a two-sample Student test, AD patients and HC and used them to radiomic analysis. Pearson’s correlation coefficients were regarded to select effective features associated with the clinical cognitive scales. Using a support vector machine as test classifier, an accuracy of 91.5% was obtained for classifying AD vs. HC.

Our study aimed to compare and contrast the strengths and limitations of radiomics and 3D deep learning CNN in extracting meaningful information from FDG PET whole brain images in order to select normal and demented subjects.

## Materials and methods

### Image acquisition

All patients who underwent 18F-FDG PET brain scans at MultiMedica Hospital were in a resting state, following ≥ 4–6 –h fast, and had a measured blood glucose level < 11.0 mmol/l at the time of study. A 222–296 MBq injection of 18F-FDG was administered intravenously under standardized conditions (in a quiet, dimly lit room with the patient’s eyes open). A 10-min three-dimensional brain emission scan was acquired at 30–45 min post-injection with a PET scanner (Siemens Biograph 8 HD PET/CT; Siemens, Germany). During the scanning procedure, the patient’s head was immobilized using a head holder. Attenuation correction was performed using low-dose computed tomography prior to the emission scan. Following corrections for scatter, dead time, and random coincidences, PET images were reconstructed using OSEM iterative reconstruction providing 110 contiguous transaxial slices of 3.3-mm-thick spacing.

### Image preprocessing

All the original Digital Imaging and Communications in Medicine data were converted into NIfTI formatted files. [18F]FDG PET images of patients and controls were normalized to the optimized [18F]FDG PET template [9], using SPM12 (https://www.fil.ion.ucl.ac.uk/spm/) implemented in MATLAB R2022b. They were then scaled to the global mean of the activity within the brain and finally smoothed with an isotropic 3D Gaussian kernel (8 mm FWHM), accordingly with the validated pipeline proposed for our single-subject SPM-based analysis [10]. This kind of smoothing is required for the random field theory to be applicable. It is also effective in reducing the number of multiple comparisons to be performed. The normalized images had a spatial resolution of 79 × 95 × 69 with voxel sizes of 2 × 2 × 2 mm3, in order to obtain rotationally invariant texture features.

**Fig. 1:**
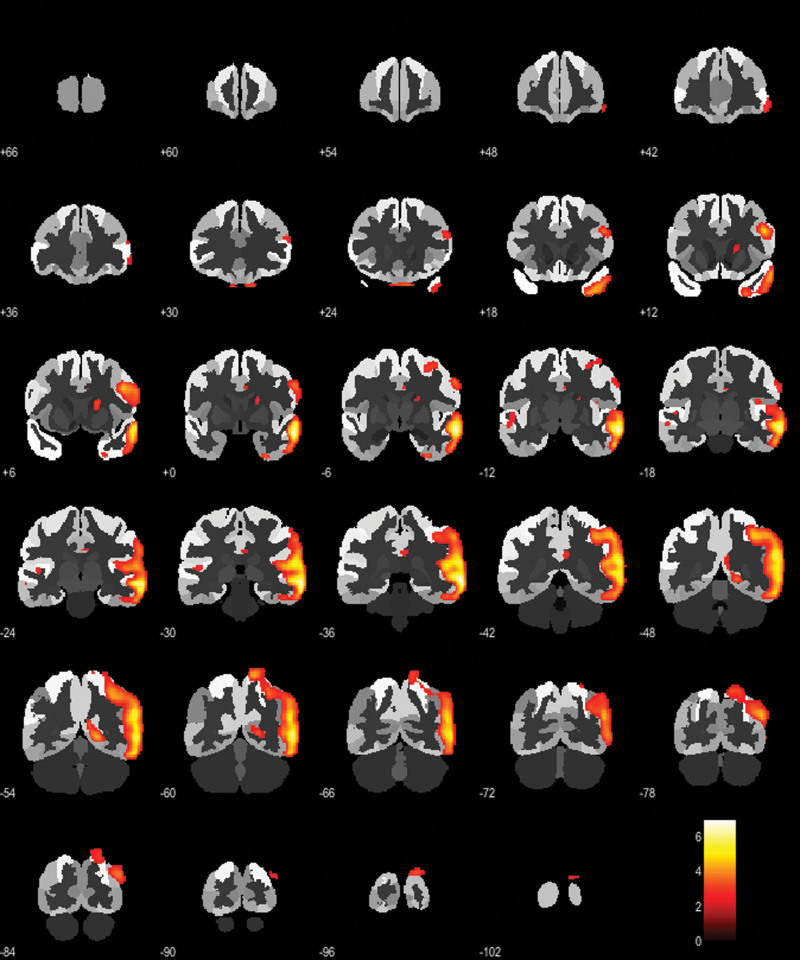
Coronal images show the result of the two-sample Student’s test to assess the difference between AD patient and HC group. Regions of significant decreases in FDG uptake are overlapped on the standard SPM template.

The SPM statistical voxel-wise procedure consists in a t test, in which a single individual is compared with a dataset of healthy control (HC), entering age as a covariate. This statistical comparison provides t-scores for each brain voxel [11]. SPM t-maps were generated from this statistical comparison in order to identify eventual brain areas of significant hypometabolism (*P* < 0.01). Only clusters containing more than 100 voxels were considered to be significant [11]. Two neuroimaging experts visually inspected all the SPM t-maps in order to confirm the presence of hypometabolism patterns compatible with neurodegenerative processes. Figure 1 shows an example of a patient with Alzheimer’s disease.

### Feature extraction

Unlike other authors [9] who used several ROIs positioned in specific areas of the brain, we preferred to employ the entire brain volume as reference (VOI). The image intensity level was discretized into 32 bins.

We computed 79 first-order statistic features (morphology, statistical, histogram, and intensity-histogram features), describing the distribution of voxel intensities within the ROI mask, and, to quantify intra-ROI heterogeneity, we extracted 136 three-dimensional textural features by analyzing the gray-level co-occurrence matrix (GLCM), run-length matrix (GLRLM), size-zone matrix (GLSZM, GLDZM) and dependence matrix (NGLDM, NGTDM).

GLCM captures spatial relationships of pairs of pixels or voxels with predefined gray-level intensities, in different directions (horizontal, vertical, or diagonal for a 2D analysis or 13 directions for a 3D analysis), and with a predefined distance between the pixels or voxels. GLCM features include entropy, a measure of gray-level inhomogeneity or randomness; angular second moment (also called uniformity or energy), which reflects gray-level homogeneity or order; and contrast, which emphasizes gray-level differences between pixels or voxels belonging to a pixel or voxel pair.

The GLRLM provides information about the spatial distribution of runs of consecutive pixels with the same gray level, in one or more directions. GLRLM features include fraction, which assesses the percentage of pixels or voxels within the ROI that are part of the runs and therefore reflects graininess; long- and short-run emphasis (inverse) moments, which are weighted toward the presence of numbers of long and short runs, respectively; and gray-level and run-length non-uniformity, which assesses the distribution of runs over different gray levels and run lengths, respectively.

The GLSZM is based on a similar principle to the GLRLM, but spatial frequency of intensity changes, so that an ROI comprising many small areas with markedly different gray levels will have greater busyness. The NGLDM is also based on the gray-level relationship between a central pixel or voxel and its neighborhood. Here, a neighboring pixel or voxel within a predefined distance is regarded as being connected to the central pixel or voxel if it meets the dependence criterion in terms of a defined range of gray-level differences. The ROI is then analyzed for the presence of central pixels or voxels with intensity i- and j-dependent neighboring pixels or voxels. Again, similar to GLRLM, NGLDM features include a large dependence emphasis and a small dependence emphasis that reflect heterogeneity and homogeneity, as well as gray-level non-uniformity and dependence uniformity that reflect the similarity in gray levels and in gray-level dependencies here, counts of the number of groups (so-called zones) of inter-connected neighboring pixels or voxels with the same gray level form the basis for the matrix. A more homogeneous texture will result in a wider and flatter matrix. GLSZM is not computed for different directions but may be computed for different pixel or voxel distances that define the neighborhood. GLSZM features may be calculated in 3 dimensions (26 neighboring voxels) and, following GLRLM definitions, include fraction (percentage of pixels or voxels that are part of the zones), large- and small-zone emphasis, and others. As a variation of GLSZM, GLDZM not only assesses zones of interconnected neighboring pixels or voxels with the same gray level but requires them to be at the same distance from the ROI edge. GLDZM features are therefore ‘“hybrids”’ between texture features and morphologic features, as is also reflected by some self-explanatory GLDZM feature names, such as small-distance high–gray-level emphasis.

Neighborhood Gray-Tone Difference Matrix (NGTDM) quantifies the sum of differences between the gray level of a pixel or voxel and the mean gray level of its neighboring pixels or voxels within a predefine distance. Key features include NGTDM coarseness, busyness, and complexity. Coarseness reflects the gray-level difference between the central pixel or voxel and its neighborhood and thus captures the spatial rate of changes in gray-level intensities; that is, an ROI consisting of larger areas with relatively uniform gray levels (i.e. a lower rate of spatial intensity changes) will have a high coarseness value. Busyness, on the other hand, reflects rapid gray-level changes between the central pixel or voxel and its neighbors.

The Standardized Environment for Radiomics Analysis (SERA) Package [12] (a Matlab-based framework) was used for this purpose, in which features are consistent with the guidelines of Image Biomarker Standardization Initiative. This package has been assessed in multi-center standardization studies [13,14] to ensure reproducibility of the features.

### Statistical analysis

**Fig. 2:**
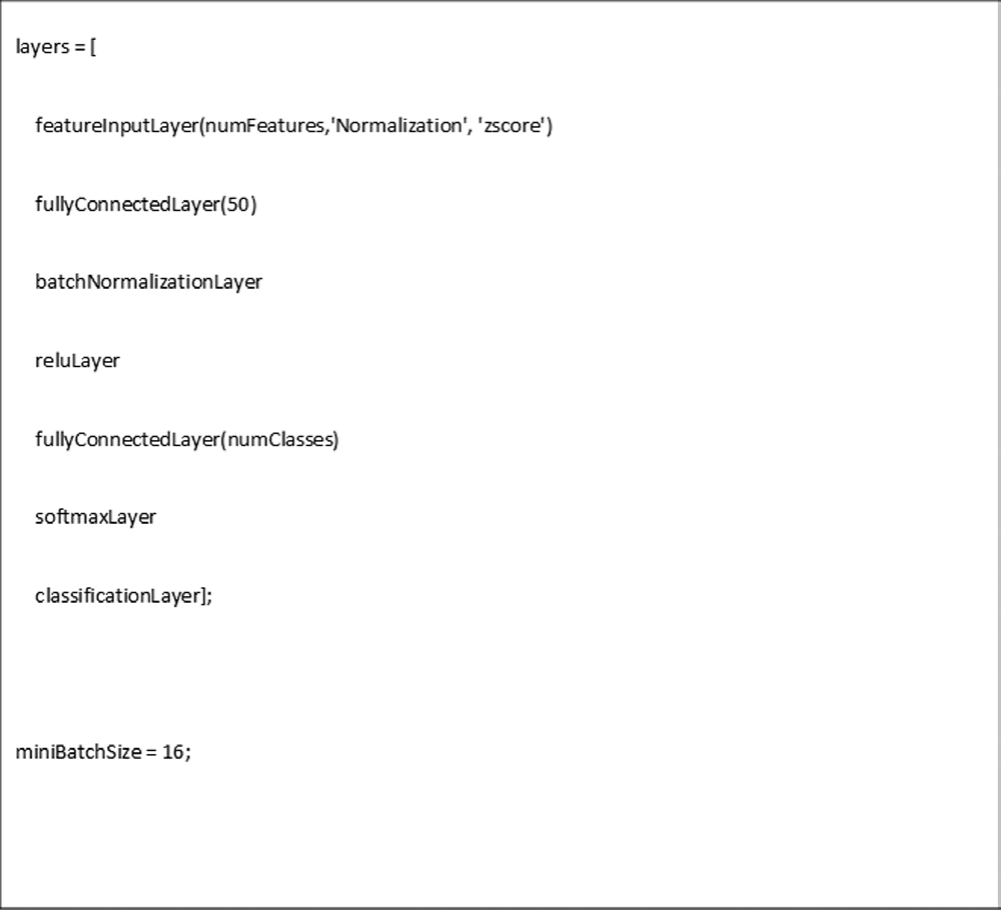
Neural network-RADIOMICS architecture.

For radiomic features analysis, the minimum redundancy maximum relevance (MRMR) method was used to address the dimensionality problem [15]. The MRMR algorithm finds an optimal set of features that is mutually and maximally dissimilar and can represent the response variable effectively. The algorithm minimizes the redundancy of a feature set and maximizes the relevance of a feature set to the response variable. Correlation analysis among the selected features was then performed in terms of Pearson correlation coefficient, selecting only those with a correlation inferior to 30%. The final set of features was used to train. For this purpose, the dataset was split into 90% training and validation data and 10% testing data. A binary classification model (Fig. 2) was built using the Matlab function ‘*featureInputLayer*’ which inputs feature data to a Neural Network and applies z-score normalization. The endpoint was to discriminate normal controls and demented patients. The accuracy of the trained model was then assessed on the testing data (20 subjects).

### CNN architecture

**Fig. 3:**
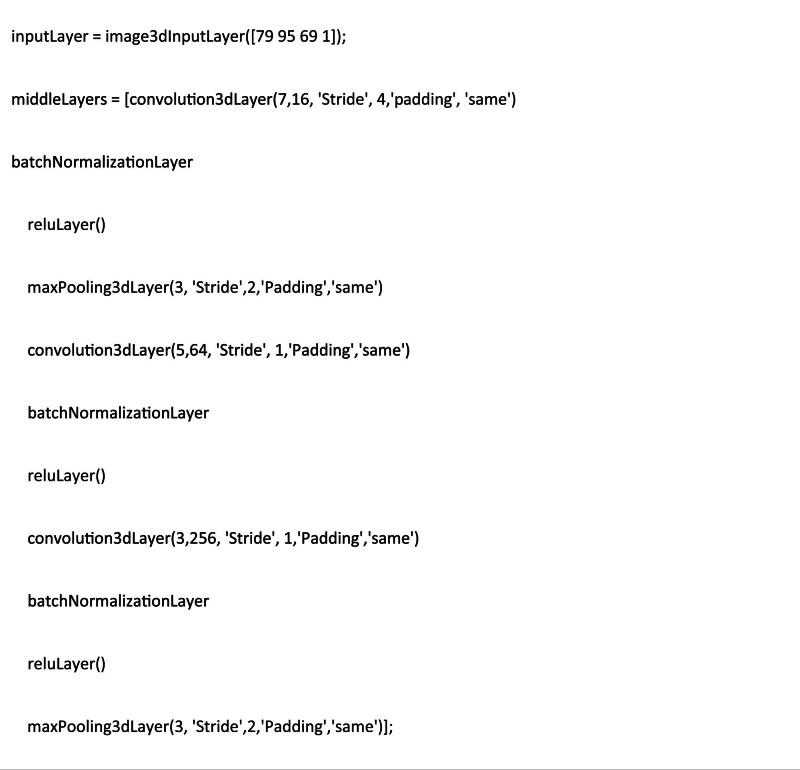
3D-CNN architecture.

CNNs are the basis of deep learning methods which excel at pattern recognition. The CNN is designed to better retain and utilize the structural information among neighboring voxels and to require minimal preprocessing by directly taking three-dimensional (3D) images as inputs. Structurally, a CNN is a sequence of layers, and each layer of the CNN transforms one volume of activations to another through a differentiable function. A typical CNN consists of three types of neural layers: convolutional layers, pooling layers and fully connected layers. The convolutional layers are interspersed with pooling layers, eventually leading to the fully connected layers. The convolutional layer takes the voxels of a small patch of the input images, called the local receptive field and then utilizes various learnable kernels to convolve the receptive field to generate multiple feature maps. A pooling layer performs the non-linear down-sampling to reduce the spatial dimensions of the input volume for the next convolutional layer. The fully connected layer input the 3D feature map to a 1D feature vector. The major issue in training deep models is the over-fitting, which arises from the gap between the limited number of training samples and a large number of learnable parameters. The batch normalization it is one of the most used methods to remedy it. The architecture of our deep 3D CNNs denoted with the sizes of input, convolution, max pooling and output layers and the numbers and sizes of generated feature maps is reported in Fig. 3. The code was in-house developed using MATLAB 2022b environment. We used 80% of the dataset for the training cohort, 10% of the dataset for the validation cohort, and 10% of the dataset for the test cohort (the same subjects used to test the radiomic model). The accuracy and the classification scores (the classification score represents the posterior probability that the individual case belongs to the class of normal or pathological) were compared with those obtained by radiomic analysis using a Student’s *t*-test (statistically significant *P* < 0.05).

### Patient population

The study was approved by the ethics committee of MultiMedica Hospital, Sesto San Giovanni, Italy. All patients of MultiMedica Hospital provided written informed consent. The data used in this study included 125 HC obtained from the AIMN website (aimn.it). The AIMN represents the Italian scientific reference for Nuclear Medicine and Molecular Imaging activities. This database comprised subjects aged between 23 and 84 years (mean: 64.8 ± 11.33). These subjects were selected because they were characterized by absence of global cognitive impairment, as assessed by a MMSE score ≥ 28, and were cognitively normal after an average 4-year clinical follow-up. From a clinical cohort referred to the Departments of Neurology and Nuclear Medicine Unit of MultiMedica Hospital (Milan, Italy), we retrospectively selected 85 consecutive patients diagnosed with probable dementia based on [18F] FDG PET and clinical examination results, namely, 38 cases diagnosed with probable Alzheimer disease (AD), 2 MCI, converted to AD [16], 25 with probable Lewy Body Dementia (DLB) [17], 20 fulfilling current clinical criteria for Frontotemporal Dementia (FTD) [18]. All patients had to have a confirmed neurological diagnosis after at least 2-year follow-up. The diagnosis was further confirmed by comparison with HC, using Student’s *t*-test as described in ‘Image Preprocessing paragraph.’ The characteristic metabolic patterns present in various causes of dementia have been reported in several previous studies [19,20]. This group comprised subjects aged between 56 and 88 years (mean: 73.9 ± 6.8), 50 males (59%). The mean Mini-Mental State Examination score was 20.2.

## Results

### Radiomic analysis

The MRMR analysis identified 21 features, equal to 10% of the total. The correlation analysis identified 4 non-collinear features (Table 1) which were used to train. Maximum and Interquartile range are simple first-order statistics features, while Dependence count non-uniformity (DN) and Low dependence low gray level emphasis (SDLGLE) belong to the category of Gray Level Dependence Matrix (GLDM), high-order features. A Gray Level Dependence Matrix (GLDM) quantifies gray level dependencies in an image, that is, the number of connected voxels within distance δ that are dependent on the center voxel. A neighboring voxel with gray level j is considered dependent on center voxel with gray level i if |i − j|≤α. In a gray level dependence matrix P (i, j) the (i, j) ^th^ element describes the number of times a voxel with gray level i with j dependent voxels in its neighborhood appears in image. Specifically, DN measures the similarity of dependence throughout the image, with a lower value indicating more homogeneity among dependencies in the image, while SDLGLE measures the joint distribution of small dependence with lower gray-level values [21].

**Table 1:** Correlation analysis among the selected features performed in terms of Pearson correlation coefficient

Features	Maximum	Low dependence low gray level emphasis	Dependence count non-uniformity	Interquartile range
Maximum	1	−0.1489	−0.2330	−0.0365
Low dependence low gray-level emphasis	−0.1489	1	0.2994	−0.2223
Dependence count non-uniformity	−0.2330	0.2994	1	−0.2239
Interquartile range	−0.0365	−0.2223	−0.2239	1

AD, Alzheimer's dementia; FT, fronto-temporal dementia; DLB, Lewy body dementia; MCI, mild cognitive impairment.

The overall validation accuracy of both models (CNN and Radiomics) was 100%. Both models correctly identified the 10 normal subjects and the 10 demented patients which constitute the test group. As shown in Table 2, the scores indicating the likelihood of normality or disease in each subject were, on average, higher using Radiomics than the CNN model (mean: 98.05 ± 2.08 vs. 85.78 ± 5.95 and mean: 97.54 ± 2.98 vs. 95.57 ± 3.92 respectively). This difference was statistically significant (*P* = 0.0004) only in the group of healthy subjects.

**Table 2:** Probability score for a subject to be normal or pathological for various types of dementia

	Patients with dementia		Health controls	
Diagnosis	Radiomics	CNN	Radiomics	CNN
FT	100	99.7	99.6	77.3
DLB	92.3	98.7	97.9	93.5
FT	98.6	98.7	99.3	86.8
MCI	93.9	91.8	94.1	97
FT	99.5	96.8	97.6	88.4
AD	99.5	98.2	100	85
FT	98.4	91.7	95.5	81.1
FT	99.9	98	96.7	80.9
AD	99.5	94	100	83.7
FT	93.8	88.1	99.8	84.1
Mean	97.54	95.57	98.05	85.78
SD	2.98	3.92	2.08	5.95
Student’s *t*-test	***P* = NS**		***P* = 0.0004**	

## Discussion

The main objective of our study was to accurately select the population of subjects with dementia through clinical evaluation, follow-up, and, above all, comparison with a group of HC. The comparison was performed using a two-sample Student’s *t*-test on each voxel, taking age into account. To simplify and replicate the analysis, all brain volumes obtained through FDG-PET were normalized using a reference template, the same one used to derive the radiomic features. The choice was dictated by two reasons. First to avoid the mispositioning of the ROIs due to the low spatial resolution of the PET. When placing the boundaries of the region or volume of interest, it needs to be ascertained that these boundaries are drawn correctly, consistently, and in a fashion that is appropriate for the research question that is to be addressed. Regions of interest should be placed by individuals well-versed in image interpretation [22,23]. Secondly, although each type of dementia has specific areas involved since the brain is an interconnected whole, an integral approach would be preferable. Furthermore, radiomic analysis applied to the entire brain volume would make it more comparable with 3D-DL approach. Due to the limited size of the dementia population, the analysis was conducted on the entire sample without dividing it based on the type of dementia observed. The purpose of the study was to compare the accuracy of the two classification models, not to evaluate their ability to differentiate between different pathological forms. To avoid overfitting in the radiomic analysis, four features were selected through an initial screening using a well-established selection program (MRMR), and subsequently, features showing significant correlation with each other were discarded. The four final selected features were used for training and validation using a standard Neural Network learning program. Concurrently, normalized 3D PET images of all patients and of the HC group were included for training and validation of a 3D-Deep Learning CNN program. The accuracy resulted in 100% for both evaluation and control group (10 HCs and 10 dementia patients) for both models. The accuracy result was predictable considering the careful selection of demented patients. In the light of current knowledge, it does not appear that other authors have carried out a similar study. The most significant finding of our research, was that the disease probability scores or normality scores obtained through Radiomics were higher compared to the CNN model in both populations, reaching high significance in the HC group. The explanation is not apparent and requires confirmation on a larger sample, preferably obtained from different centers. It is an area of discussion whether CNNs or Radiomics are preferable in the field of medical imaging and neuroradiology. A major incentive for favoring Radiomics is the black-box nature of CNNs—the basis of the output of the CNN is not always readily explicable. In a radiomic pipeline, features are extracted based on predefined mathematical equations, designed by image analysis experts. In our study, the most specific parameters in order to distinguish normal from pathological subjects were probably the two high-order features. Compared to the Student’s *t*-test analysis, which simply identify groups of voxels whose counts are below the normality threshold, radiomics allows us to assess the homogeneity or inhomogeneity of distribution of these voxels, improving our ability to discriminate normal from demented subjects. Claiming to distinguish the various types of dementia solely based on the characteristic metabolic patterns it appears too simplistic. Often the various types of dementia, especially at the onset of the disease, show superimposable metabolic patterns; in these cases, the texture analysis should improve the diagnostic specificity.

However, not every radiomic pipeline is easily comprehensible either. The interpretation of second and higher-order radiomic features can be quite challenging. Some feature reduction methods such as principle component analysis, on the other hand, may fuse different features in a non-reversible way. Finally, the model itself may not be transparent. If a feed-forward neural network is used, for example, it can be difficult to determine how much each feature contributed to the output [23].

### Limitations

There are several caveats when conducting Radiomics and DL research. The small size of our demented group was only partially compensated by the high-quality images obtained with homogeneous acquisition protocols and reconstruction techniques. Furthermore, radiomic features are notorious to be significantly sensitive to batch effect (variability in imaging acquisition parameters in different centers, scanner models and reconstruction settings) [24]. A number of harmonization methods have been proposed to correct for the batch effect to generate robust and reproducible models. But these methods are mandatory when using multi-center datasets [25], while our patients come from only one. The next goal of our research group will be to confirm the results on a larger sample of patients, comparing the different forms of dementia and to include some clinical parameters such as MMSE in the training.

### Conclusion

The novel approach based on high-order radiomic features extracted from standardized 18F-FDG PET whole brain images seems to be more accurate than deep learning 3D approach to distinguish patients with and without dementia.

## Acknowledgements

The authors wish to recognize the valuable human and professional contribution of technical and nursing staff of Nuclear Medicine Department (Angela Lepre, Donatella Alberti, Paolo Sciaresa, Krisztina Hosszù). We are also grateful to Mr. Giovanni Maria Bestetti for his invaluable assistance in this study.

### Conflicts of interest

There are no conflicts of interest.

## Supplementary Material
